# Obesity surgery and risk of colorectal and other obesity-related cancers: An English population-based cohort study

**DOI:** 10.1016/j.canep.2018.01.002

**Published:** 2018-04

**Authors:** Ariadni Aravani, Amy Downing, James D. Thomas, Jesper Lagergren, Eva J.A. Morris, Mark A. Hull

**Affiliations:** a Leeds Institute of Cancer & Pathology, University of Leeds, Worsley Building, Leeds, LS2 9NL, United Kingdom; b Public Health England, Blenheim House, Duncombe St., Leeds, West Yorkshire, LS1 4PL, United Kingdom; c Upper Gastrointestinal Surgery, Department of Molecular Medicine and Surgery, Karolinska Institutet, Karolinska University Hospital, 17176 Stockholm, Sweden; d Division of Cancer Studies, King’s College London, WC2R 2LS, United Kingdom; e Leeds Institute of Biomedical & Clinical Sciences, University of Leeds, Wellcome Trust Brenner Building, St. James’s University Hospital, Leeds LS9 7TF, United Kingdom

**Keywords:** CRC, colorectal cancer, OS, obesity surgery, RYGB, Roux-en-Y gastric bypass, HES, Hospital Episode Statistics, ONS, Office of National Statistics, SIR, standardised incidence ratio, NCRAS, National Cancer Registration & Analysis Service, Bariatric surgery, Colon cancer, Rectal cancer, Obesity

## Abstract

**Background:**

The association between obesity surgery (OS) and cancer risk remains unclear. We investigated this association across the English National Health Service. A population-based Swedish study has previously suggested that OS may increase the risk of developing colorectal cancer (CRC).

**Methods:**

A retrospective observational study of individuals who underwent OS (surgery cohort) or diagnosed with obesity, but had no OS (no-surgery cohort) (1997–2013) were identified using Hospital Episode Statistics. Subsequent diagnosis of CRC, breast, endometrial, kidney and lung cancer, as well as time ‘at risk’, were determined by linkage to National Cancer Registration & Analysis Service and Office of National Statistics data, respectively. Standardised incidence ratios (SIR) in relation to OS were calculated.

**Results:**

1 002 607 obese patients were identified, of whom 3.9% (n = 39 747) underwent OS. In the no-surgery obese population, 3 237 developed CRC (SIR 1.12 [95% CI 1.08–1.16]). In those who underwent OS, 43 developed CRC (SIR 1.26 [95% CI 0.92–1.71]). The OS cohort demonstrated decreased breast cancer risk (SIR 0.76 [95% CI 0.62–0.92]), unlike the no surgery cohort (SIR 1.08 [95% CI 1.04–1.11]). Increased risk of endometrial and kidney cancer was observed in surgery and no-surgery cohorts.

**Conclusions:**

CRC risk is increased in individuals diagnosed as obese. Prior obesity surgery was not associated with an increased CRC risk. However, the OS population was small, with limited follow-up. Risk of breast cancer after OS is reduced compared with the obese no-surgery population, while the risk of endometrial and kidney cancers remained elevated after OS.

## Highlights

• The association between obesity surgery (OS) and cancer risk remains unclear.
• Colorectal cancer (CRC) risk is increased in obese individuals.
• No evidence of increased CRC risk after OS in this English population study.
• Reduced breast cancer risk was apparent after OS.
• Renal and endometrial cancer risk is elevated in obese patients.

## 1 Introduction

Obesity is linked to an increased risk of several malignancies, including colorectal (CRC) [[1], [2], [3]] post-menopausal breast [[4], [5], [6]], endometrial [[7], [8]] and kidney cancers [[9], [10]]. Obesity (also known as bariatric) surgery (OS) is an effective treatment for weight reduction providing metabolic and cardiovascular benefits[11]. In parallel with the increased prevalence of obesity, there has been a significant increase in the frequency of OS [12]. Traditional OS procedures such as gastric banding and Roux-en-Y gastric bypass (RYGB), which induce weight loss via restrictive and combined restrictive/malabsorbtive mechanisms respectively, are the most commonly performed worldwide [11]. Over the last decade, sleeve gastrectomy has emerged as an alternative procedure [[11], [13]].

The effect of OS on future risk of CRC is not clear. Counterintuitively, there is evidence that OS may increase the long-term risk of developing CRC despite post-operative weight loss [[14], [15], [16], [17]]. The effect appears to be time-dependent, with the risk of CRC increasing with time from surgery, which would be consistent with the long natural history of colorectal carcinogenesis. It is plausible that colorectal carcinogenesis may be driven by changes in diet and the gut microbiota post-bariatric surgery [[18], [19]]. By contrast, a meta-analysis of four observational studies, which have reported CRC incidence after OS, concluded that overall OS is associated with a 27% lower risk of subsequent CRC [20].

However, all studies to date, except one population-based Swedish study [14] have been limited in their follow-up time after OS (less than ten years) and sample size (so statistical power) to fully explore the association with incident CRC [[21], [22], [23]]. We aimed therefore, to confirm or refute the findings of the Swedish study in a separate independent population. We tested the hypothesis that there is an increase in CRC incidence following OS in a large population-based cohort of individuals who had undergone OS in England, also determining the risk of other obesity-related cancers for comparison.

## 2 Methods

### 2.1 Design

This was a national population-based retrospective observational data-linkage study of individuals over the age of 18 and below 95 years, who had an episode of in-patient or day-case care in an English NHS hospital involving a primary diagnosis of obesity or OS. Study approval was obtained from the Health Research Authority Confidentiality Advisory Group (CAG) (CAG reference: CAG 4-09(b)/2013) and Research Ethics Committee (REC reference: 13/YH/0204). This research was funded by World Cancer Research Fund International (WCRF) and Cancer Research UK (CRUK).

Patients diagnosed with obesity were identified using the International Classification of Diseases Version 10 (ICD10): E66 code. OS was defined as an episode of care with a primary diagnosis of obesity with an Office of Population Censuses and Surveys (OPCS) Classification of Interventions and procedures (4th revision) procedure code for a surgical procedure listed in Table 1. These individuals were identified using a Hospital Episode Statistics (HES) dataset containing hospital admissions between April 1997 and September 2013. We reviewed OPCS4 codes used by NHS Digital (previously the Health and Social Care Information Centre) in previous analyses and excluded several procedures that were either; 1) very unlikely to be performed as OS, or 2) were a revision, reversal or maintenance procedure [[24], [25]]. Table 1 details the codes used by NHS Digital and the codes used in this study. If individuals within this cohort had multiple episodes of care of the same type recorded (OS or obesity without surgery), then the first episode of care took precedence. If an individual had both OS and obesity no surgery episodes recorded then the surgery episode was used.

**Table 1:** Office of Population Censuses and Surveys 4th revision (OPCS4) codes defined as obesity surgery (OS) by NHS Digital and this study.

3-Digit OPCS4 Code	4-Digit OPCS4 Code	Description	NHS Digital	This Study
G01	G011 – G019	excision of oesophagus and stomach	*	
G02	G021 – G029	total excision of oesophagus	*	
G03	G031 – G039	partial excision of oesophagus	*	
G27	G271 – G279	total excision of stomach	*	
G28	G281	partial gastrectomy and anastomosis of stomach to duodenum	*	
	G282	partial gastrectomy and anastomosis of stomach to transposed jejunum	*	*
	G283	partial gastrectomy and anastomosis of stomach to jejunum NEC	*	*
	G284	sleeve gastrectomy and duodenal switch	*	*
	G285	sleeve gastrectomy NEC	*	*
	G288	other specified partial excision of stomach	*	*
	G289	unspecified partial excision of stomach	*	*
G30	G301	gastroplasty not elsewhere classified	*	*
	G302	partitioning of stomach	*	*
	G303	partitioning of stomach using band	*	*
	G304	partitioning of stomach using staples	*	
	G305	maintenance of gastric band	*	
	G308	other specified plastic operation on stomach	*	*
	G309	plastic operation on stomach NOS	*	*
G31	G310 – G319	connection of stomach to duodenum	*	
G32	G320	conversion from previous anastomosis of stomach to transposed jejunum	*	
	G321	bypass of stomach by anastomosis of stomach to transposed jejunum	*	*
	G322	revision of anastomosis of stomach to transposed jejunum	*	*
	G323	conversion to anastomosis of stomach to transposed jejunum	*	*
	G324	closure of connection of stomach to transposed jejunum	*	
	G325	attention to connection of stomach to transposed jejunum	*	*
	G328	other specified connection of stomach to transposed jejunum	*	*
	G329	unspecified connection of stomach to transposed jejunum	*	*
G33	G330	conversion from previous anastomosis of stomach to jejunum NEC	*	
	G331	revision of anastomosis of stomach to jejunum NEC	*	*
	G332	conversion to anastomosis of stomach to jejunum nec	*	*
	G333	closure of connection of stomach to jejunum NEC	*	*
	G334	open reduction of intussusception of gastroenterostomy	*	
	G335	closure of connection of stomach to jejunum	*	
	G336	attention to connection of stomach to jejunum	*	*
	G338	other specified other connection of stomach to jejunum	*	*
	G339	unspecified other connection of stomach to jejunum	*	*
G38	G387	removal of gastric band	*	
	G388	other specified	*	*
G48	G481 – G486	other operations on stomach	*	
G49	G491 – G499	excision of duodenum	*	
G51	G511	bypass of duodenum by anastomosis of stomach to jejunum	*	*
	G513	bypass of duodenum by anastomosis of duodenum to jejunum	*	
G61	G611	bypass of jejunum by anastomosis of jejunum to jejunum		*
	G619	bypass of jejunum by anastomosis of jejunum to colon		*
G71	G716	duodenal switch		*

The cohort was linked to the National Cancer Registration & Analysis Service (NCRAS) dataset to determine if these individuals received, subsequent to the index episode (OS or obesity alone), a diagnosis of CRC (ICD10 C18-C20), breast (ICD10 C50), kidney (ICD10 C64) or endometrial (ICD10 C54) cancer, which are all cancers known to be linked to obesity [[14], [16], [26]]. In contrast, lung cancer (ICD10: C33-C34) is not obesity-related [26] but was included as a control as its incidence should be unaffected by OS. Lastly, upper gastrointestinal cancers (esophageal cancer (ICD-10: C15), stomach cancer (ICD-10: C16), small intestine cancer (ICD-10: C17), liver cancer (ICD-10: C22), gallbladder cancer (ICD-10: C23), extrahepatic bile duct cancer (ICD10: C24) and pancreatic cancer (ICD10: C25)) were included in the data as the codes used to identify OS are similar to those used for surgical procedures used to manage these cancers. Individuals with upper gastrointestinal cancers were subsequently excluded from the analyses.

The cohort was linked to the Office for National Statistics (ONS) mortality dataset to determine individual time at risk of cancer diagnosis. This was defined as the time from the index episode to cancer diagnosis, death or the censor date (30th September 2013).

The characteristics of the groups who did and did not undergo OS, subsequently referred to as surgery and no-surgery cohorts, were compared. This revealed a relatively high proportion of individuals that apparently underwent OS a short period after a diagnosis of cancer. These operations were likely to be associated with cancer management rather than to treat obesity. Thus, all individuals who developed a cancer within one year of the index episode were excluded.

### 2.2 Statistical analysis

The standardized incidence ratio (SIR) with 95% confidence interval (CI) was calculated as an estimate of relative risk of both surgery and no-surgery obese participants diagnosed with a cancer instead of making a direct comparison between the two cohorts that could be confounded by differences in age, calendar year and other risk factors. The SIR was calculated as the ratio of the observed number of cancer cases in the study population to the number that would be expected if that population experienced the same cancer incidence rates as the background English population, dependent on age and calendar period. This was achieved by splitting follow-up time into one-year age categories and one-year calendar periods and each age-period-sex group was then linked with cancer incidence rates in England obtained from NCRAS. The expected number of cancer cases was calculated for both the surgery and no-surgery cohorts by multiplying the observed person time by age, sex and calendar year-specific cancer incidence rates for England. The follow-up time after OS was classified as: 1 to 2 or ≥2 years. All person-time during the first year after surgery or diagnosis of obesity was excluded because of the risk of erroneous identification of procedures associated with cancer resection or palliation, rather than OS, or earlier detection of CRC due to hospitalization or obesity surgery. This widened exclusion by reducing all individuals’ risk time by one year, and not only those who were diagnosed with cancer within one year from the index event. Finally, the observed and expected numbers of deaths were summed and divided. The SIR with 95% CI was estimated under the assumption that the observed number of events followed a Poisson distribution.

## 3 Results

### 3.1 Patients

**Fig. 1:**
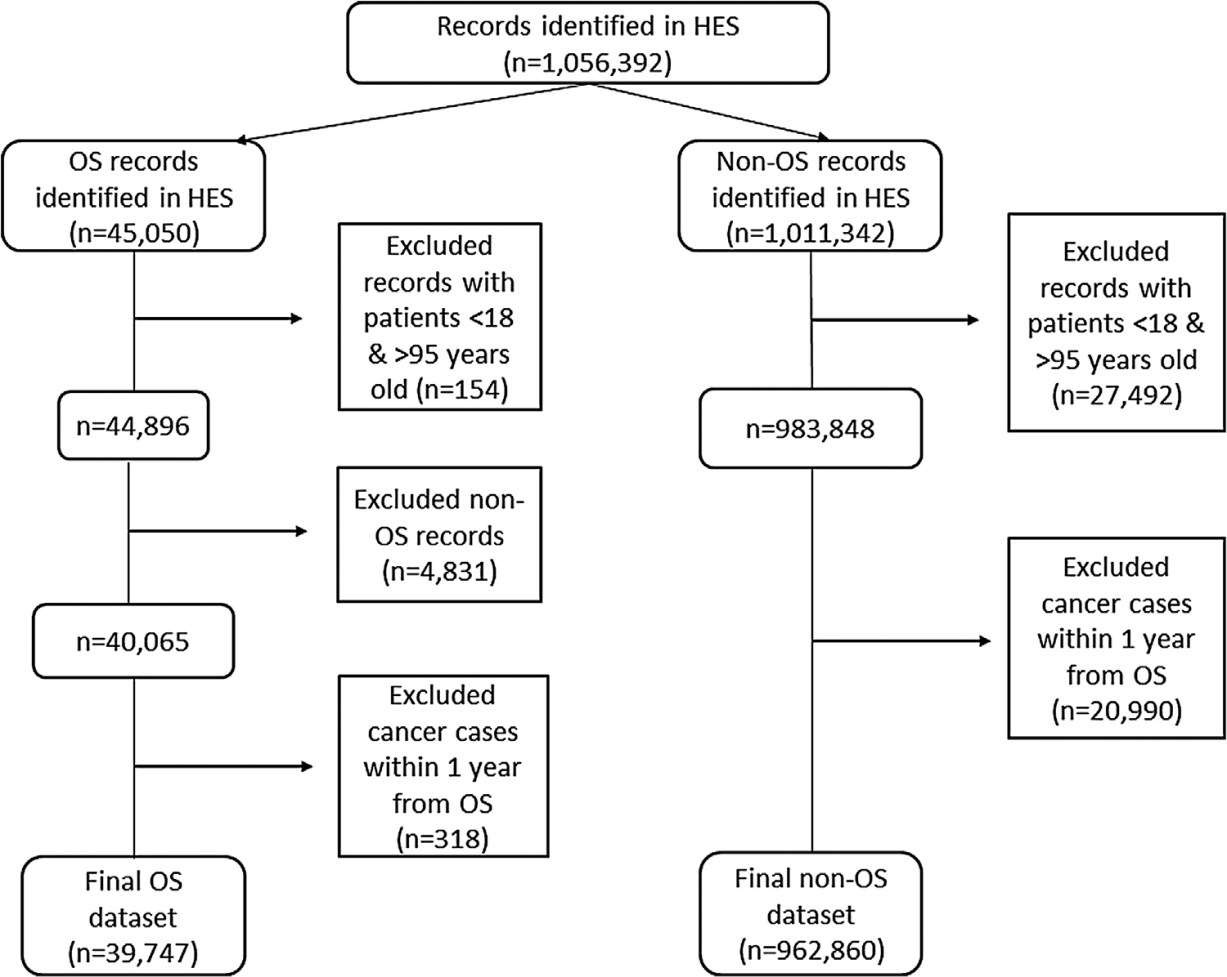
Record identification and exclusions from the Hospital Episode Statistics (HES) datasets for the obesity surgery (OS) and obese no OS groups.

A total of 1 056 392 patients were initially identified. After exclusions, the final dataset consisted of 1 002 607 individuals, including; 39 747 (3.9%) recorded as having OS and the remainder (962 860; 96.0%) as having an episode of hospital care due to obesity without OS (Fig. 1). Table 2 details the characteristics of the two groups. The majority of patients in both groups were female; 76.6% in the OS group and 62.9% in the obese no OS group. The OS group was younger than the no surgery group, with a mean age of 44.8 and 53.1 years, respectively. The majority of OS (91.7%) took place after 2006 and this restricted the potential follow-up time after surgery to six years for the majority of this population. The OS group had a median follow-up period of 3.0 years (range 1–16 years) and 144 677 person-years of follow-up. The equivalent figures for the obese no OS group were a median follow-up time of 2.5 years (range 1–16 years) and 3 608 882 person-years at risk.

**Table 2:** Characteristics of the obesity surgery (OS) group and the obese no OS group identified in HES between 1997 and 2013 excluding all cancers diagnosed within 1 year from index episode.

	OS, number (%)		Obese no OS, number (%)	
Total	39,747	100.0%	962,860	100.0%
Gender				
Male	9311	23.4%	356,855	37.1%
Female	30,436	76.6%	606,005	62.9%
Age groups at entry into the cohorts, years				
18–39	12,552	31.6%	247,032	25.7%
40–49	13,957	35.1%	154,197	16.0%
≥50	13,238	33.3%	561,631	58.3%
Calendar year				
1997–2005	3282	8.3%	168,684	17.5%
2006–2013	36,465	91.7%	794,176	82.5%
Follow-up time, years				
≤2	13,021	32.8%	402,879	41.8%
>2	26,726	67.2%	559,981	58.2%
Surgery type				
Restrictive surgery	20,649	52.0%	–	–
Restrictive and malabsorbtive surgery	19,098	48.0%	–	–

### 3.2 Risk of colorectal cancer

There were 43 new diagnoses of CRC in the OS group and 3 237 new diagnoses in the obese no OS group. Table 3 shows the SIR for CRC diagnosis in the two groups, after exclusion of all person-time within one year from the OS surgery or hospital attendance associated with obesity. Comparisons were not made directly between the two groups, but between each group and the English background population. The absolute cumulative incidence of CRC in the surgery group was lower (30 per 100 000 person-years) than that in the no surgery group (91 per 100 000 person-years), which is likely explained by the younger age of the surgery cohort. The overall SIR for CRC in the surgery cohort was not significantly increased compared to the background English population (SIR: 1.26 95% CI: 0.92–1.71). There was, however, a slightly increased CRC risk in individuals who did not undergo surgery (SIR: 1.12, 95% CI: 1.08–1.16) compared to the background population. In the surgery group, an increased risk of CRC was observed in the oldest (≥50 years) age group (SIR: 1.47, 95% CI: 1.02–2.06). In the no-surgery group, the SIR of CRC was higher in males (SIR: 1.21, 95% CI: 1.15–1.26) than in females (SIR: 1.02, 95% CI: 0.97–1.08). The risk of CRC was higher in the no-surgery group in the latest calendar period (2006–2013) (SIR: 1.16, 95% CI: 1.12–1.21) compared to the earliest period (1995–2005) (SIR: 0.94, 95% CI: 0.86–1.02).

**Table 3:** Risk of colorectal cancer expressed as the standardized incidence ratio (SIR) with 95% confidence interval (lower confidence limit (LCL), upper confidence limit (UCL)) in the obesity surgery (OS) and the obese no OS groups.

Variable	OS				Obese No OS			
	*n*	SIR	LCL	UCL	*n*	SIR	LCL	UCL
Total	43	1.26	0.92	1.71	3237	1.12	1.08	1.16
Gender								
Male	16	1.41	0.81	2.29	1850	1.21	1.15	1.26
Female	27	1.19	0.79	1.74	1387	1.02	0.97	1.08
Age groups at entry into the cohorts, years								
18–39	2	0.79	0.10	2.85	54	1.35	1.02	1.77
40–49	7	0.83	0.34	1.72	174	1.19	1.02	1.38
≥50	34	1.47	1.02	2.06	3009	1.11	1.07	1.15
Calendar year								
1997–2005	5	2.17	0.70	5.07	497	0.94	0.86	1.02
2006–2013	38	1.20	0.85	1.65	2740	1.16	1.12	1.21
Follow-up time, years								
1–2	26	1.24	0.81	1.81	1706	1.14	1.09	1.20
≥2	17	1.32	0.77	2.11	1531	1.10	1.04	1.15
Surgery type								
Restrictive surgery	29	1.41	0.94	2.02	–	–	–	–
Restrictive and malabsorptive surgery	14	1.05	0.57	1.76	–	–	–	–

### 3.3 Risk of other obesity-related and non-obesity-related cancers

Table 4 shows the SIR of breast, endometrial or kidney cancer (obesity-related cancers) and lung cancer (not obesity-related) diagnosis. There was an overall decreased risk of breast cancer after OS (SIR: 0.76, 95% CI: 0.62–0.92), and slightly increased risk in obese individuals who did not undergo OS (SIR: 1.08, 95% CI: 1.04–1.11). The risk of endometrial cancer was increased by nearly 3-fold for both groups (SIR: 2.98, 95% CI: 2.25–3.90, for surgery and SIR: 2.60, 95% CI: 2.48–2.73, for no-surgery groups) compared with the background population. The risk of kidney cancer was increased approximately 3-fold after OS (SIR: 3.06, 95% CI: 2.08–4.34) and almost 2-fold in obese individuals who did not undergo OS (SIR: 1.78, 95% CI: 1.68–1.89). The risk of lung cancer was, however, reduced in the surgery group (SIR: 0.70, 95% CI: 0.46–1.03) while it was slightly raised in the no-surgery group (SIR: 1.09, 95% CI: 1.05–1.13).

**Table 4:** Standardized incidence ratios (SIRs) with 95% confidence intervals (lower confidence limit (LCL), upper confidence limit (UCL)) for non-colorectal cancers in the obesity surgery (OS) and the obese no OS groups.

Cancer site	OS				Obese No OS			
	*n*	SIR	LCL	UCL	*n*	SIR	LCL	UCL
Breast	101	0.76	0.62	0.92	3806	1.08	1.04	1.11
Uterus	54	2.98	2.25	3.90	1758	2.60	2.48	2.73
Kidney	31	3.06	2.08	4.34	1110	1.78	1.68	1.89
Lung	26	0.70	0.46	1.03	3645	1.09	1.05	1.13

## 4 Discussion

This study demonstrates that elevated CRC risk continues after OS, in individuals who underwent OS over the age of 50 years, an age-range at which a significant number of OS procedures are undertaken [27]. By contrast, surgery and accompanying weight loss were associated with reduced breast cancer risk, unlike the no-surgery comparator group, in which breast cancer risk was consistently elevated compared with the background population. There is limited literature available examining the association between obesity surgery and cancer risk. Two Swedish studies [[14], [16]], showed an association between bariatric surgery and increased risk of obesity-related cancers during long-term follow-up, whilst other studies with limited power to interpret CRC-specific risk have shown the opposite phenomenon [[20], [21], [23], [28]].

Methodological strengths of this study include the population-based cohort design, which reduced selection bias and the large size of the cohort. Another significant strength of this study is the high completeness and validity of the linked HES and NCRAS datasets used in previous studies [[29], [30], [31], [32], [33]]. Studies using these data have shown that the final linked dataset is stronger and richer than its component parts (cancer registration and hospital admissions) with robust recording of cancer incidence [[34], [35]]. In addition the process of linkage enables the identification of duplicates in both datasets, so their combination improves the overall quality of the data available.

There are, however, several important limitations of the data, upon which the study was based, which should be highlighted. The obese no surgery cohort represents a small subset of the obese UK population, since it includes those individuals who have been admitted or had an outpatient or A&E appointment related to obesity. The study was originally intended to replicate a previous population-based Swedish study [14], which showed an association between OS and increased risk of CRC, using a larger English population and, hence, with greater statistical power. Whilst the initial extract of data, however, comprised 1 002 606 individuals (13-times bigger than the Derogar et al. study), the number of individuals in the OS group was significantly lower (4% of the total obesity population), as the OS rates in England have increased only recently in response to increased obesity incidence. Additionally, the limited follow-up time in this study was likely not sufficient for a statistically significant association of OS with CRC risk to be revealed (median follow-up three years). On the contrary, in the Swedish study OS patients were followed-up for a considerably longer period of time (mean follow-up time nine years) allowing a statistically significant association to be revealed.

Another limitation of this study is the fact that no definitive list of OPCS4 codes for OS exists and other studies have used different codes [[24], [25], [36]]. The codes used in this study were based on those used in previous NHS Digital reports [[24], [25]] excluding some codes in order to make our analysis more specific. For instance, oesophagogastrectomy and anastomosis of oesophagus to stomach (OPCS 4: G011) was excluded as it was anticipated that such an extensive procedure including the extraction of oesophagus and stomach was probably not intended to be for obesity management. Another example of a code that we excluded is conversion from previous anastomosis of stomach to duodenum (OPCS 4: G310). We excluded 4865 cases, anticipated not to be related to OS, which led to a smaller (but more specific) cohort than originally anticipated. In addition, the codes in HES for OS overlap with procedures for some cancer procedures. At the outset this was not anticipated to influence our cohort significantly as only episodes of care with a primary diagnosis of obesity (and not cancer) were included. However, the data extract that we received from NHS Digital did contain a large number of OS procedures that were undertaken around the time of the diagnosis of a cancer and these were assumed to be surgery linked to cancer treatment. Exclusion of these cases necessarily reduced the size of the cohort available for subsequent analysis.

Sensitivity analyses were conducted using the full list of OS procedures used by NHS Digital in previous publications [[24], [25]]. This did not significantly affect the results. Although there were marked differences between the list used by NHS Digital and the one used in our analysis, the number of procedures identified with sufficient follow-up to influence the results were small. In consequence, little difference was observed. Once further follow-up data are available then such analyses would be more revealing.

Furthermore, some of the codes used to indicate OS (notably sleeve gastrectomy (G285) and gastric banding (G303)) were not introduced into the OPCS4 coding system until April 2006. It was not clear, prior to this date, what codes were being used in HES for these procedures. This resulted in fewer ‘bariatric’ procedures being identified in the 9 years of the study period prior to their introduction, and significantly more subsequently. This had the effect of both further reducing the size of the cohort but also, importantly, limiting the follow-up time for the OS group. The unexpectedly small sample having undergone OS was another difference from the previous Swedish study [14]. In that study, 20% of the population underwent OS compared to only 4% in the current study. Indeed, Sweden has the second highest rate of use and the highest levels of spending per capita on OS in Europe [37], while England has the fifth highest rate. In addition, the clinical indications for use of the surgery vary between the two countries with Sweden offering the procedure to all at a body mass index (BMI) of 40 kg/m^2^ with no serious co-morbidities [37], whilst English patients are also required to have an undergone an intensive weight loss programme for at least 12–24 months (6 months for those with BMI >50 kg/m^2^) prior to surgery. Evidence suggests that OS is used in Sweden for individuals with lower BMIs and less comorbidity than in England [37].

Our population-based linkage study did not include data on BMI and other risk factors for CRC [38]. Data on BMI after OS, obtained through linkage to primary care data are required to investigate further the association between OS and CRC risk in the UK. Such data would also provide the opportunity to perform a preliminary investigation of whether colorectal adenoma risk increases after OS, as benign colorectal adenoma is a recognized biomarker of CRC risk [39] allowing a shorter follow-up period in a cohort study. Other risk factors such as ethnicity, socioeconomic status or insurance status were not available in this dataset.

It should be recognized that patients undergoing OS are likely to have different characteristics to those who did not have surgery, including age and presence of obesity-related co-morbidities. These characteristics are also associated with the incidence of obesity-related cancers. In addition, obese people who do not have OS may be at a higher risk of non-cancer related morbidity and mortality [[40], [41], [42]]. Again, this may influence our findings by leading to a higher censoring rate, and so shorter follow-up time, in the non-surgery versus the OS cohort. This bias could not be taken into account due to insufficient information in the routine data on relevant factors.

An unexpected finding was the high SIRs for kidney and endometrial cancer in both OS and obese no OS groups compared with the background population. We do not believe that this is explained by erroneous coding of cancer-related surgery as all cancer diagnoses occurring within one year after the diagnosis of obesity were excluded and the SIR was significantly above unity even in the obese no OS group. Both cancers are recognized as having a strong association with excess body weight [[7], [8], [9]]. The SIR that we observed for endometrial cancer is similar to the relative risk associated with increasing body mass index in the UK Million Women study [43]. However, a recent systematic review and meta-analysis has suggested that endometrial cancer risk is reduced after OS [44]. Subsequent lung cancer risk was lower in those who underwent OS compared with the obese no OS group which likely reflects non-smoker selection bias for OS. Previously, it has been reported that excess body weight is protective for lung cancer, especially in current and former smokers [[26], [45]].

### 4.1 Conclusions

In conclusion, using a population-based data linkage approach, we report increased CRC risk in individuals diagnosed as obese. Although the interpretation of whether OS is associated with subsequent higher CRC risk was limited by the small OS group size and restricted follow-up time after OS, the data indicated statistically significant increased SIRs in obese patients older than 50 years at the time of the surgery. This could be due to longer exposure to obesity before OS, something that requires further investigation. We also report that OS is associated with reduced breast cancer risk, unlike the obese comparator group. Finally, we report high SIRs for renal and endometrial cancers in the presence or absence of prior OS, which warrants further investigation.

## Author contribution statement

EJAM, MAH, AD, JL contributed on the study design and conception. JDT carried out the linkage to the National Cancer Registration and Analysis Service and extracted the data. AA carried out the analysis and AA, EJAM, MAH, JL and AD contributed to the interpretation of the data and results of the analyses. AA, EJAM, MAH, JL and AD contributed drafting the manuscript and critically revising it. All authors contributed to the final version. All authors approved the final version to be published.

## Funding

This study was funded by WCRF (2012/596) and CRUK (C23434/A23706).

## Conflict of interest statement

No conflict of interest.
